# Initiation and Persistence of Pharmacotherapy for Youths with Attention Deficit Hyperactivity Disorder in Taiwan

**DOI:** 10.1371/journal.pone.0161061

**Published:** 2016-08-12

**Authors:** Liang-Jen Wang, Kang-Chung Yang, Sheng-Yu Lee, Chun-Ju Yang, Ting-Shuo Huang, Tung-Liang Lee, Shin-Sheng Yuan, Yu-Chiau Shyu

**Affiliations:** 1 Department of Child and Adolescent Psychiatry, Kaohsiung Chang Gung Memorial Hospital and Chang Gung University College of Medicine, Kaohsiung, Taiwan; 2 Genome and Systems Biology Degree Program, National Taiwan University and Academia Sinica, Taipei, Taiwan; 3 Institute of Statistical Science, Academia Sinica, Taipei, Taiwan; 4 Department of Psychiatry, Kaohsiung Veterans General Hospital, Kaohsiung, Taiwan; 5 Department of Psychiatry, College of Medicine and Hospital, National Cheng Kung University, Tainan, Taiwan; 6 Institute of Biopharmaceutical Sciences, National Yang-Ming University, Taipei, Taiwan; 7 Community Medicine Research Center, Keelung Chang Gung Memorial Hospital, Keelung, Taiwan; 8 Department of General Surgery, Keelung Chang Gung Memorial Hospital, Keelung, Taiwan; 9 Department of Chinese Medicine, College of Medicine, Chang Gung University, Taoyuan, Taiwan; 10 Department of Experimental Radiation Oncology, University of Texas MD Anderson Cancer Center, Houston, Texas, United States of America; 11 Institute of Molecular Biology, Academia Sinica, Nankang, Taipei, Taiwan; Philipps-Universitat Marburg, GERMANY

## Abstract

**Background:**

Pharmacotherapy is an effective therapeutic option for attention deficit hyperactivity disorder (ADHD). Understanding the patterns of medication treatment is crucial for clinical practice. This study employed nationwide population-based data to elucidate the initiation and persistence of pharmacotherapy (immediate-release methylphenidate [IR–MPH], osmotic controlled-release formulations of methylphenidate [OROS–MPH] and atomoxetine [ATX]) for youths with ADHD in Taiwan.

**Methods:**

Patients first receiving an ADHD diagnosis at age 18 or younger between January 2000 and December 2009 (n = 112,140; mean age at ADHD diagnosis: 7.7 years) were selected from Taiwan’s National Health Insurance database. All patients were monitored through December 31, 2011, with an average follow-up time of 5.8 years. The initiation of ADHD drug therapy was defined as the first patient prescription, and discontinuation was defined as the cessation of ADHD medication for 180 days or longer.

**Results:**

Within the first year after ADHD diagnosis, 47.3%, 14.4%, and 0.8% of the patients were prescribed IR–MPH, OROS–MPH, and ATX, respectively. Regarding the patients prescribed IR–MPH, OROS–MPH, and ATX, 17.8%, 12.6%, and 18.8%, respectively, received the prescription only once and never returned for a drug refill, and 51.0%, 38.9%, and 58.8%, respectively, discontinued drug therapy within 1 year after the first prescription. Male sex and neuropsychiatric comorbidities were associated with higher probabilities of being prescribed one of the medications. An older age at first prescription and a higher daily dose of prescription were significant predictors of early discontinuation of ADHD medication.

**Conclusions:**

The current findings suggest that IR–MPH is the most frequently prescribed drug for ADHD treatment in Taiwan. Patients treated with OROS–MPH possessed the highest persistence rate, whereas those treated with ATX had the lowest persistence rate. The results provide insight into the delivery of pediatric mental health services and have crucial implications for ADHD medication treatment in real clinical settings.

## Introduction

Attention deficit hyperactivity disorder (ADHD) is a common neurodevelopmental disorder that begins in childhood [1]. It affects approximately 5% to 7% of school-age children worldwide [2, 3], and a prevalence rate of 7.5% was reported in a study of Taiwan [4]. The core symptoms of ADHD are inattention, hyperactivity, and impulsivity [5]. These symptoms may cause a lack of expected progress in academic achievement, interpersonal relationships, and family or social interaction [6, 7]. Pharmacotherapy is an effective therapeutic option and has been suggested as the first-choice ADHD treatment [8]. Two main classes of medications, namely stimulants and nonstimulants have been approved for ADHD treatment [9], though different medications and preparations are available in various countries [10].

Although compelling evidence has demonstrated the effectiveness of ADHD pharmacotherapy [11], public controversies regarding the safety and necessity of ADHD drug therapy have continued for decades [12–14]. A previous survey showed that only approximately 30% of participants were willing to provide medication to a child with ADHD [15]. Several large population studies have examined the proportion of ADHD patients receiving drug therapy. For example, Chen et al. [16] reported that approximately 30% of young people in Taiwan received methylphenidate (MPH) treatment within 1 year of ADHD diagnosis. Chien et al. [17] revealed that the proportion of MPH use in youths with ADHD increased from 39.6% in 1997 to 54.0% in 2005. Winterstein et al. [18] indicated that utilization of pharmacologic treatment ranged from 2.5% to 4.6% for youths in the US Medicaid database. McCarthy et al. [19] reported the annual incidence of pharmacological ADHD treatment was 1.28–1.45 per 1000 among general population in the UK between 2003 and 2008. Garbe et al. [20] revealed that 52% of children and adolescents with ADHD in Germany received ADHD drug treatment. Zoega et al. [21] reported that the prevalence of ADHD medication use in 2007 among the total Nordic population was 2.76 per 1000 inhabitants. A Danish cohort showed that 80% of patients who were prescribed ADHD medications used only MPH, whereas 6% used only atomoxetine [22].

ADHD medications in routine clinical care are effective only when drug therapy is administered to the patient persistently. Premature discontinuation of ADHD medication may result in inadequate treatment responses and negative long-term outcomes [23, 24]. A pharmacoepidemiological study conducted in UK indicated that the rate of patients maintaining ADHD drugs therapy largely fell short of the estimated rate of ADHD [25]. A systematic review of literature published between 1990 and 2013 [26] was employed to analyze the existing knowledge regarding ADHD medication discontinuation. Treatment persistence of stimulants, measured by treatment duration during 12-month follow-up periods, averaged 136 days for youths with ADHD. One review article indicated that the average rates of nonadherence in children and adults ranged between 15% and 87% during a follow-up period up to 9 years [27]. More recent data reported by Zetterqvist et al. [28] suggested that patients in Sweden aged 15–21 were the most likely to discontinue treatment; after 3 years and 11 months, 27% of those patients were still receiving treatment. Among children and adolescents with ADHD in Turkey, medication persistence over a continuous 12-month period occurred at a rate of 30.2% [29]. Lambert et al. [30] reported that the median treatment duration for psychostimulants was 1.96 years in a state in Australia from 1990 to 2010.

Prior to 2011 in Taiwan, immediate-release methylphenidate (IR–MPH), osmotic controlled-release formulation of methylphenidate (OROS–MPH), and atomoxetine (ATX) were the only three drugs approved for ADHD treatment [31]. IR–MPH and OROS–MPH are short-acting (half-life: 2–3 h) and extended-release stimulants (8–12 h), respectively [32]. Taiwan’s National Health Insurance Administration has suggested IR–MPH as the first-line drug therapy for ADHD, and OROS–MPH for patients who experience poor drug compliance or are intolerant of the adverse effects of IR–MPH. ATX is a selective norepinephrine reuptake inhibitor that is classified as a nonstimulant [33] and is a second-line medication in the management of ADHD in children who are unresponsive to stimulants, or for patients with comorbid ADHD and tic or anxiety disorders. Understanding the patterns and factors that affect initiation and persistence in ADHD medication treatment may improve treatment efficacy and symptom control. However, few studies have compared data regarding various MPH formulations and ATX separately [20, 34–36].

Therefore, this study employed nationwide population-based data to elucidate the prescription patterns (initiation and persistence) of pharmacotherapy (IR–MPH, OROS–MPH and ATX) for youths with ADHD in Taiwan, and investigated the factors associated with these patterns.

## Methods

### Ethical Statement

The protocol for this study conformed to the Helsinki Declaration, and was approved by the Institutional Review Board (IRB) of Chang Gung Memorial Hospital. Patient records/information was anonymized and de-identified prior to analysis, and the need for written informed consent was waived by the IRB.

### Data Source

Data for this study were obtained from the ambulatory claims database of the National Health Insurance Research Database (NHIRD). Implemented in 1995, Taiwan’s National Health Insurance (NHI) program is a compulsory universal health insurance program. The Bureau of National Health Insurance is the single payer for healthcare services under the NHI, and has contracted 93% of all healthcare providers in Taiwan. More than 96% of insured people have used healthcare services at least once in contracted hospitals and clinics since 1995. Contracted medical care institutions must submit monthly medical expense-related claim documents electronically, comprising patient demographic data, medical institutions visited, diagnostic codes, dates of prescriptions, drugs prescribed, and claimed medical expenses. Validation studies have demonstrated that the coding in the NHIRD system is a valid resource for population research [37].

### Definition of ADHD Patients and Comorbidities

We included all patients with records in the NHIRD who were aged 18 years or less and newly diagnosed with ADHD between January 2000 and December 2009. To reduce the possibility of misdiagnosis, an ADHD patient was defined as a patient having at least two NHI claim records in accordance with International Classification of Diseases, Ninth Revision, Clinical Modification (ICD-9-CM) code 314.00 (attention deficit disorder without mention of hyperactivity) or 314.01 (attention deficit disorder with hyperactivity). Finally, our study sample comprised 112,140 patients with ADHD. Patient NHIRD medical records were followed up through December 31, 2011; thus, patients had a follow-up period ranging from 2 to 12 years (mean duration: 5.8 ± 2.7 years).

The following relevant neurodevelopmental disorders that are commonly comorbid with ADHD were examined in this study and grouped as follows: oppositional defiant disorder (ODD) or conduct disorder (CD) (ICD-9-CM code 313.81 or 312.X), autistic spectrum disorder (ASD) (ICD-9-CM code 299.X), tic disorder (ICD-9-CM code 307.2X), and intellectual disability (ICD-9-CM codes 317–319), anxiety disorders (ICD-9-CM codes 300.X) and depressive disorders (ICD-9-CM codes 296.2, 296.3, 300.4, and 311).

### Definition of Initiation and Discontinuation of Drug Therapy

Pharmacotherapies for ADHD were identified using the anatomical therapeutic chemical classification system. According to the Food and Drug Administration of Taiwan, only three drugs were licensed for treating ADHD before 2011: IR–MPH (ATC code N06BA04), OROS–MPH (ATC code N06BA04), and ATX (ATC code N06BA09). IR–MPH entered the market in Taiwan on March 1, 1995; OROS–MPH entered the market on September 1, 2004; and ATX entered the market on May 1, 2007. Any prescription of IR–MPH, OROS–MPH, or ATX was recorded using an ambulatory care claim, pharmacy claim, or hospital care claim.

The initiation of ADHD drug therapy was defined as first patient prescription of IR–MPH, OROS–MPH, or ATX. We defined the index date as the date that the ADHD medication was first prescribed, and patient NHIRD medical records were followed up through December 31, 2011 or until discontinuation of medication treatment. The discontinuation of ADHD medication was defined as cessation of IR–MPH, OROS–MPH, or ATX for 180 days or longer. We defined the discontinuation date as the date of the last prescription administered before drug cessation. The total follow-up duration was defined as the duration between the index date and the discontinuation date or December 31, 2011. The average daily doses of ADHD medications were calculated based on the defined daily dose suggested by the WHO Collaborating Centre for Drug Statistics Methodology [38].

### Statistical Analysis

All statistical analyses were performed using the Statistical Package for Social Sciences (SPSS) Version 21.0 (SPSS Inc., Chicago, IL, USA). Variables were expressed as mean (standard deviation), median (range), or frequency values. A two-tailed *P* value of < 0.05 indicated statistical significance. A chi-squared (χ^2^) test or *t* test was applied to compare the characteristics of the ADHD patients who were prescribed ADHD medications and those who were not.

We employed a multivariate logistic regression model to estimate the potential factors associated with drug therapy initiation, adjusted for the year of ADHD diagnosis. The factors associated with treatment with IR–MPH, OROS–MPH, and ATX were analyzed separately. The adjusted odds ratios and 95% confidence intervals (CI) were calculated. Survival curves were employed to express the probability of initiating ADHD drug therapy over time.

In addition, Cox regression models [39] were fitted to the data to assess the potential factors for drug therapy discontinuation. In the survival analysis, the time function was determined as the number of days from initial observation to December 31, 2011 (end of monitoring). The monitoring time was determined as the time between the index date of ADHD drug therapy and the date of drug discontinuation (or until the end of the study period for censored patients). The adjusted hazard ratio and 95% CI were calculated. Survival curves were also used to express the time function of ADHD drug therapy discontinuation.

## Results

Of the 112,140 youths (mean age at ADHD diagnosis: 7.7 ± 3.1 years; 20.7% female) included in this study, 63.9% were prescribed ADHD medication (IR–MPH, OROS–MPH, or ATX) at least once during the study period. Furthermore, 35.9%, 46.0%, 49.1%, and 54.0% of ADHD youths received treatment of any of the three drugs within 30 days, 180 days, 1 year, and 2 years of their ADHD diagnosis, respectively. Compared with the unmedicated ADHD patients (**Table 1**), the patients who received drug therapy were older (mean age at ADHD diagnosis: 8.3 ± 3.0 years), had a higher proportion of males (81.7%), and were more likely to have an ODD/CD (12.0%), tic disorder (6.8%), intellectual disability (15.1%) or anxiety disorder (31.8%) comorbidity. Of all the patients, 61.6%, 32.0%, and 4.7% were prescribed at least one dose of IR–MPH, OROS–MPH, and ATX during the study period, respectively.

**Table 1:** Characteristics of youths newly diagnosed with ADHD (N = 112,140) between January 2000 and December 2009 in Taiwan.

Characteristics	Total (N = 112140)	With drug therapy (N = 71666)	Without drug therapy (N = 40474)	Statistical value
**Sex**				
Female	23206 (20.7)	13095 (18.3)	10111 (25.0)	χ^2^ = 709.05***
Male	88934 (79.3)	58571 (81.7)	30363 (75.0)	
**Age at diagnosis of ADHD (years)**	7.7 ± 3.1	8.3 ± 3.0	6.7 ± 2.9	*t* = 83.99***
**Psychiatric comorbidity**				
ODD or conduct disorder	10533 (9.4)	8635 (12.0)	1898 (4.7)	χ^2^ = 1645.30***
Autistic spectrum disorder	11464 (10.2)	7328 (10.2)	4136 (10.2)	χ^2^ = 0.001
Tic disorder	6962 (6.2)	4901 (6.8)	2061 (5.1)	χ^2^ = 135.20***
Intellectual disability	15539 (13.9)	10824 (15.1)	4715 (11.6)	χ^2^ = 258.22***
Anxiety disorders	27703 (24.7)	22758 (31.8)	4945 (12.2)	χ^2^ = 5308.20***
Depressive disorders	5089 (4.5)	3298 (4.6)	1791 (4.4)	χ^2^ = 1.867
**Drug therapy for ADHD**				
Any medication	71666 (63.9)	71666 (100)	0 (0)	—
IR–MPH	69110 (61.6)	69110 (96.4)	0 (0)	—
Average daily dose (mg)	15.1 ± 8.3	15.1 ± 8.3	0 (0)	—
OROS–MPH	35922 (32.0)	35922 (50.1)	0 (0)	—
Average daily dose (mg)	26.1 ± 8.9	26.1 ± 8.9	0 (0)	—
ATX	5283 (4.7)	5283 (7.4)	0 (0)	—
Average daily dose (mg)	30.3 ± 11.4	30.3 ± 11.4	0 (0)	—
**Year of recruitment**				χ^2^ = 930.63***
2000 to 2001	11598 (10.3)	8430 (11.8)	3168 (7.8)	
2002 to 2003	13510 (12.0)	8949 (12.5)	4561 (11.3)	
2004 to 2005	21876 (19.5)	14307 (20.0)	7569 (18.7)	
2006 to 2007	30256 (27.0)	19622 (27.4)	10634 (26.3)	
2008 to 2009	34900 (31.1)	20358 (28.4)	14542 (35.9)	
**Duration of follow-up (days)**	2125.3 ± 986.4	2190.5 ± 998.1	2009.8 ± 954.3	*t* = 29.57***

*Note*: Data are expressed as mean ± SD or n (%); IR–MPH: immediate-release methylphenidate; OROS–MPH: osmotic controlled-release formulation of methylphenidate; ATX: atomoxetine

***P < 0.001.

Among the patients who received pharmacotherapy, 93.9%, 5.4%, and 0.7% were started on IR–MPH, OROS–MPH, and ATX, respectively. Among the patients who received prescriptions for IR–MPH, 46.8% switched to OROS–MPH, and 6.7% switched to ATX. In addition, 93.7% of the patients who received OROS–MPH were prescribed IR–MPH in the past, and 93.8% of the patients who received ATX treatment were prescribed IR–MPH or OROS–MPH in the past.

### Initiation of Drug Therapy

For patients who were prescribed IR–MPH, OROS–MPH, or ATX (**Table 2**), the age at first prescription was 9.0 ± 2.8, 10.3 ± 2.7, and 10.9 ± 2.9 years, respectively; the mean time from ADHD diagnosis to drug prescription was 286.5 ± 549.7, 760.3 ± 800.6, and 1289.7 ± 919.7 days, respectively. The proportions of users of a particular drug receiving corresponding prescriptions within 30 days, 180 days, 1 year, and 2 years after ADHD diagnosis were as follows: 55.6%, 71.7%, 76.8%, and 84.7% were prescribed IR–MPH; 15.4%, 35.1%, 44.9%, and 59.3% were prescribed OROS–MPH; and 4.2%, 10.5%, 17.5%, and 32.0% were prescribed ATX.

**Table 2:** Characteristics of ADHD youths prescribed IR–MPH, OROS–MPH, or ATX during the study period.

Characteristics	IR-MPH (N = 69110)	OROS-MPH (N = 35922)	ATX (N = 5283)
**Sex**			
Female	12644 (18.3)	6027 (16.8)	798 (15.1)
Male	56646 (81.7)	29895 (83.2)	4485 (84.9)
**Age at diagnosis of ADHD (years)**	8.2 ± 3.0	8.3 ± 3.0	7.4 ± 2.8
**Psychiatric comorbidity**			
ODD or conduct disorder	8396 (12.1)	5676 (15.8)	1012 (19.2)
Autistic spectrum disorder	7123 (10.3)	3860 (10.7)	948 (17.9)
Tic disorder	4622 (6.7)	2823 (7.9)	1004 (19.0)
Intellectual disability	10519 (15.2)	4941 (13.8)	849 (16.1)
Anxiety disorders	22181 (32.1)	12697 (35.3)	2305 (43.6)
Depressive disorders	4232 (6.1)	2383 (6.6)	480 (9.1)
**Initiation of treatment**			
Age at first prescription (years)	9.0 ± 2.8	10.3 ± 2.7	10.9 ± 2.9
Days between ADHD diagnosis and first prescription	286.5 ± 549.7	760.3 ± 800.6	1289.7 ± 919.7
Median (range)	21 (0–4178)	492 (0–4310)	1137 (0–4279)
Prescribed within 30 days	38447 (55.6)	5525 (15.4)	222 (4.2)
Prescribed within 180 days	49567 (71.7)	12595 (35.1)	555 (10.5)
Prescribed within 1 year	53066 (76.8)	16114 (44.9)	925 (17.5)
Prescribed within 2 years	58527 (84.7)	21300 (59.3)	1692 (32.0)
**Persistence of treatment during follow-up**			
Age at the last prescription (years)	10.6 ± 3.1	12.0 ± 3.0	11.4 ± 2.9
Days between first prescription and discontinuation	539.3 ± 714.9	444.2 ± 507.7	150.7 ± 176.6
Median (range)	193 (1–4183)	234 (1–2495)	73 (2–848)
Only received prescription once	12304 (17.8)	4530 (12.6)	991 (18.8)
Discontinued within 180 days	29173 (42.2)	10680 (29.7)	2519 (47.7)
Discontinued within 1 year	35271 (51.0)	13959 (38.9)	3106 (58.8)
Discontinued within 2 years	42562 (61.6)	18021 (50.2)	3518 (66.6)
Average daily dose (mg)	15.1 ± 8.3	26.1 ± 8.9	30.3 ± 11.4

Note: Data are expressed as mean ± SD or n (%); the three patient groups were not independent samples; 33674 (93.7%) of the patients receiving OROS–MPH treatment were prescribed IR–MPH previously; 4954 (93.8%) of the patients receiving ATX treatment were prescribed IR–MPH or OROS–MPH previously.

Regarding the factors associated with initiation of ADHD drug therapy (**Table 3**), male sex and comorbidity with ODD/CD, ASD, tic disorder, intellectual disability, or an anxiety disorder were collectively associated with a higher probability of being prescribed IR–MPH, OROS–MPH, or ATX. However, comorbidity with a depressive disorder was associated with a lower probability of being prescribed IR–MPH or OROS–MPH, but was associated with a higher probability of being prescribed ATX. Older age at ADHD diagnosis was associated with a higher probability of being prescribed IR–MPH or OROS–MPH; however, prescription of ATX was associated with younger age at ADHD diagnosis.

**Table 3:** Logistic regression models for factors associated with initiating ADHD medication treatment.

Characteristics	Any prescription	IR–MPH	OROS–MPH	ATX
	aOR (95% CI)	aOR (95% CI)	aOR (95% CI)	aOR (95% CI)
**Sex** (male vs. female)	1.49 (1.44–1.54)***	1.45 (1.40–1.49)***	1.42 (1.37–1.47)***	1.36 (1.25–1.47)***
**Age at ADHD diagnosis**	1.20 (1.20–1.21)***	1.17 (1.16–1.18)***	1.08 (1.07–1.08)***	0.94 (0.93–0.95)***
**Psychiatric comorbidity**				
ODD or conduct disorder	2.48 (2.35–2.62)***	2.36 (2.24–2.48)***	2.58 (2.48–2.69)***	2.23 (2.07–2.40)***
Autistic spectrum disorder	1.08 (1.03–1.13)***	1.06 (1.02–1.11)**	1.14 (1.09–1.19)***	1.69 (1.56–1.83)***
Tic disorder	1.22 (1.15–1.29)***	1.11 (1.05–1.17)***	1.37 (1.30–1.44)***	3.72 (3.44–4.01)***
Intellectual disability	1.48 (1.42–1.54)***	1.45 (1.39–1.50)***	1.08 (1.03–1.12)***	1.21(1.11–1.31)***
Anxiety disorders	2.97 (2.86–3.08)***	2.88 (2.78–2.98)***	2.18 (2.12–2.25)***	2.53 (2.38–2.69)***
Depressive disorders	0.66 (0.61–0.71)***	0.62 (0.58–0.66)***	0.81 (0.76–0.86)***	1.45 (1.30–1.62)***
**Year of recruitment**	0.94 (0.94–0.95)***	0.93 (0.93–0.94)***	1.04 (1.04–1.05)***	1.10 (1.09–1.11)***

*Note*: aOR: adjusted odds ratio; CI: confidence interval; ODD: oppositional defiant disorder; IR–MPH: immediate-release methylphenidate; OROS–MPH: osmotic controlled-release formulation of methylphenidate; ATX: atomoxetine

**P < 0.01

***P < 0.001.

### Persistence of Drug Therapy

**Fig 1:**
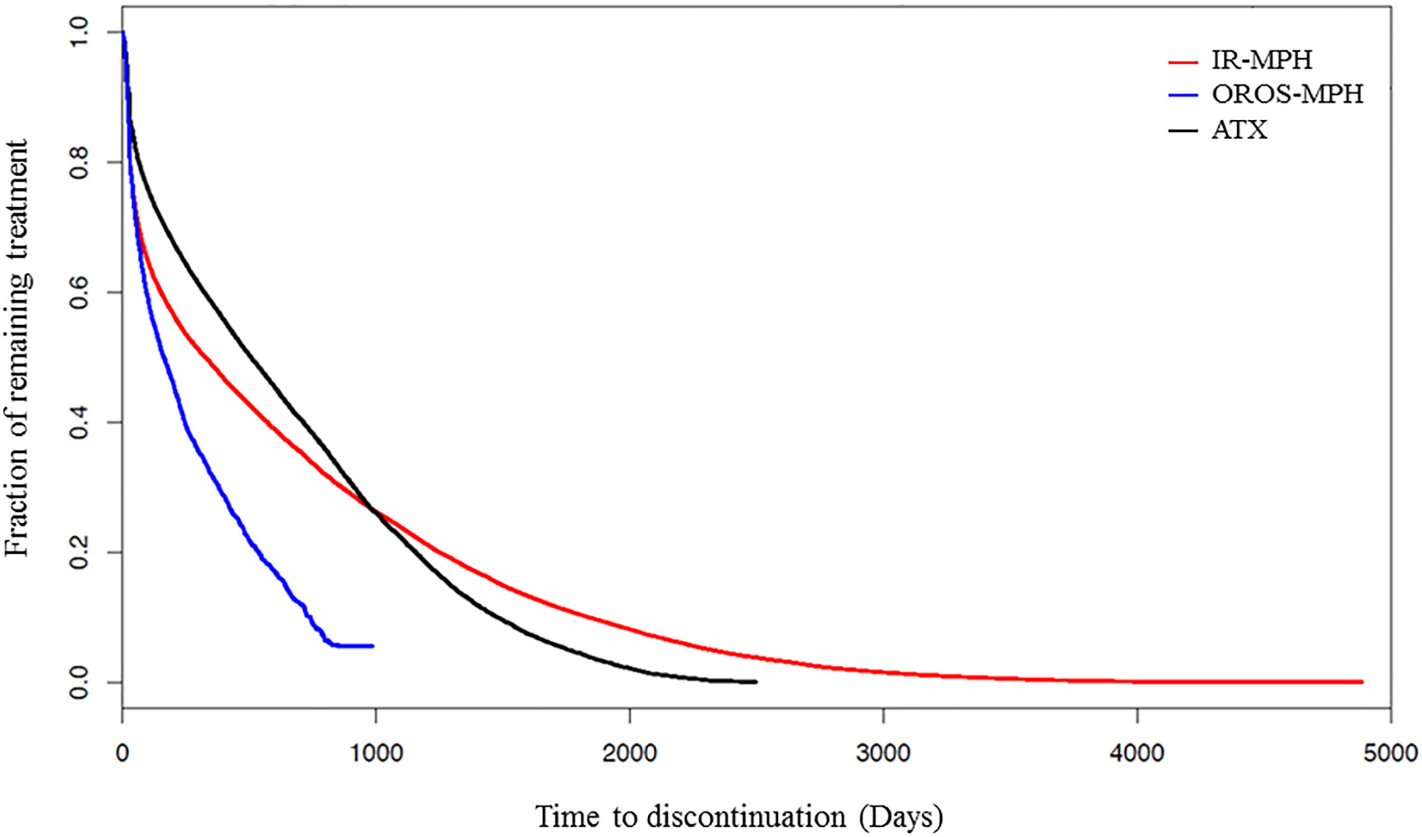
Survival curves of discontinuing treatment of immediate-release methylphenidate (IR-MPH), osmotic controlled-release formulation of methylphenidate (OROS–MPH) and atomoxetine (ATX) after ADHD patients were first prescribed these medications.

**Fig 1**shows the survival curve of discontinuing treatment of IR-MPH, OROS-MPH and ATX after ADHD patients were first prescribed these medications. During the follow-up period, 85.0%, 65.8%, and 67.4% of IR–MPH, OROS–MPH, and ATX, respectively, users discontinued their drug therapy. Among the patients prescribed IR–MPH, OROS–MPH, and ATX (**Table 2**), 17.8%, 12.6%, and 18.8%, respectively, received prescriptions only once and never returned for drug refill; 57.8%, 70.3%, and 52.3%, respectively, remained in drug treatment 6 months after first prescription; 49.0%, 61.1%, and 41.2%, respectively, remained in treatment 1 year after first prescription; and 38.4%, 49.8%, and 33.4%, respectively, remained in treatment 2 years after first prescription. Among the patients who discontinued their treatment with IR–MPH, OROS–MPH, and ATX, the age at discontinuation was 10.6 ± 3.1 years, 12.0 ± 3.0 years, and 11.4 ± 2.9 years, respectively; the mean time between drug therapy initiation to discontinuation was 539.3 ± 714.9 days, 444.2 ± 507.7 days, and 150.7 ± 176.6 days, respectively.

Among the patients who were prescribed IR–MPH (**Table 4**), female sex, older age at first prescription and higher daily dose of IR-MPH significantly predicted IR–MPH early discontinuation; whereas comorbidity for ODD/CD, ASD, tic disorder, intellectual disability, anxiety disorder, or an depressive disorder was correlated with continued IR–MPH treatment. Among the patients prescribed OROS–MPH, female sex, older age at first prescription, comorbidity with intellectual disability, and higher dose of OROS-MPH significantly predicted early discontinuation, and having ODD/CD, anxiety disorder, or a depressive disorder was correlated with persistence in drug treatment. For ATX users, older age at first prescription, depressive disorder and higher dose of ATX significantly predicted early discontinuation, and having ASD, tic disorder, intellectual disability, or an anxiety disorder was correlated with persistence in drug treatment.

**Table 4:** Cox regression models for predictors of discontinuing ADHD medication treatment during the follow-up period.

Characteristics	Any prescription	IR-MPH	OROS-MPH	ATX
	aHR (95% CI)	aHR (95% CI)	aHR (95% CI)	aHR (95% CI)
**Sex** (male vs. female)	0.87 (0.85–0.89)***	0.90 (0.89–0.92)***	0.93 (0.90–0.97)***	1.04 (0.95–1.14)
**Age at first prescription**	1.12 (1.11–1.12)***	1.11 (1.11–1.11)***	1.11 (1.11–1.12)***	1.02 (1.01–1.04)**
**Psychiatric comorbidity**				
ODD or conduct disorder	0.75 (0.73–0.77)***	0.87 (0.85–0.90)***	0.85 (0.82–0.89)***	1.00 (0.92–1.09)
Autistic spectrum disorder	0.84 (0.81–0.86)***	0.90 (0.87–0.92)***	0.97 (0.92–1.01)	0.83 (0.76–0.91)***
Tic disorder	0.82 (0.79–0.85)***	0.93 (0.90–0.96)***	0.98 (0.94–1.03)	0.82 (0.75–0.89)***
Intellectual disability	0.87 (0.85–0.90)***	0.86 (0.84–0.88)***	1.05 (1.01–1.09)*	0.88 (0.80–0.96)**
Anxiety disorders	0.83 (0.81–0.84)***	0.87 (0.85–0.88)***	0.94 (0.91–0.96)***	0.90 (0.84–0.96)**
Depressive disorders	0.82 (0.79–0.85)***	0.80 (0.77–0.83)***	0.93 (0.88–0.98)**	1.18 (1.05–1.33)**
**Average daily dose (DDD)**	1.45 (1.43–1.47)***	1.58 (1.56–1.60)***	1.22 (1.20–1.23)***	1.18 (1.07–1.29)***
**Year of ADHD diagnosis**	1.11 (1.10–1.11)***	1.13 (1.13–1.14)***	1.10 (1.09–1.11)***	1.00 (0.98–1.01)

*Note*: aHR: adjusted hazard ratio; CI: confidence interval; ODD: oppositional defiant disorder; DDD: defined daily dose; IR–MPH: immediate-release methylphenidate; OROS–MPH: osmotic controlled-release formulation of methylphenidate; ATX: atomoxetine

*P < 0.05

**P < 0.01

***P < 0.001.

## Discussion

The current study revealed that 63.9% of the patients with ADHD received pharmacotherapy at least once within an average follow-up time of 5.8 years. Because IR–MPH has been designated as the first-line medication for ADHD treatment, it was the most popular drug prescribed for ADHD youths, followed by OROS–MPH and ATX. Male sex and neuropsychiatric comorbidities were associated with higher probabilities of being prescribed one of the medications. Among the patients prescribed the three medications, those who received OROS–MPH showed the highest persistence of drug therapy during the follow-up period. Older age at first prescription and a higher daily dose of prescribed medications significantly predicted early discontinuation of the medication.

### Initiation of Drug Treatment

We found that 63.9% of patients with ADHD received at least one type of pharmacotherapy during the study period. A previous Taiwanese study [16] reported that approximately 30% of young people received MPH treatment within 1 year of their ADHD diagnosis. Furthermore, Chien et al. [17] revealed that the proportion of MPH use in youths with ADHD ranged from 39.6% to 54.0% in Taiwan from 1997 to 2005. The proportion of patients who received drug therapy in the present study was higher than that in previous studies; various methodological differences may explain this phenomenon. First, the samples used in previous studies used data collected before 2005 [16, 17, 40], rather than the most recent data. Second, our study population is comprised of all patients ever diagnosed with ADHD among the whole population of Taiwan (twenty-three million people). However, the population samples in previous studies [16, 17, 40] were ADHD patients from only one million people randomly selected from whole population in Taiwan. Therefore, compared to previous studies, our sample size is larger and is more representative for the entire population. Furthermore, the definition of ADHD patients in previous studies as any patient with at least one ADHD NHI claim record was less strict than our definition, which required at least two such claim records. Third, previous studies only analyzed the use of stimulants (MPH), and follow-up time was only 1 year. By contrast, our study conducted a comprehensive survey of stimulant and nonstimulant use for a nationwide population with a longer follow-up time (average: 5.8 years). Therefore, we suggest that our findings are more representative of the situation in Taiwan.

Because IR–MPH has been designated as the first-line medication for ADHD treatment in Taiwan, 93.9% of patients receiving ADHD pharmacotherapy were started on IR–MPH. The mean time from ADHD diagnosis to IR–MPH prescription was 286.5 days; however, 55.6% of IR–MPH users began their IR–MPH treatment within 1 month of ADHD diagnosis. Winterstein et al. [18] reported that 54.3%–58.0% of ADHD youths in the U.S. who received pharmacotherapy initiated their drug therapy within 6 months of first ADHD diagnosis. This implies that over half of the caregivers understand that ADHD is a neurobiological condition and accept medication as a valid treatment, and these patients started medication treatment within a short time after ADHD was diagnosed [41]. For other patients, pharmacotherapy may have been unacceptable, and they were typically started on rehabilitation treatment or psychosocial treatment [42]. Therefore, 46% of the ADHD youths did not receive any drug therapy or were prescribed medication for over 2 years after ADHD diagnosis. Among the patients undergoing IR–MPH treatment, 46.8% switched to OROS–MPH, and 6.7% switched to ATX. Because IR–MPH was usually prescribed prior to OROS-MPH and ATX, the average age at the first prescription of OROS–MPH and ATX was higher than that of IR–MPH.

We found that older age at ADHD diagnosis and male sex were associated with being prescribed IR–MPH or OROS–MPH. This finding is generally consistent with those of previous international studies [18, 20, 21]. However, prescription of ATX was associated with younger age at ADHD diagnosis. Among the three ADHD drugs, ATX is the newest on the market in Taiwan, and is usually prescribed as a second-line medication. Therefore, a patient diagnosed at a younger age with a longer history of medication treatment might be more likely to switch to ATX. Furthermore, we found that neurodevelopmental comorbidities were associated with stimulants treatment. This suggests that patients with comorbidities may exhibit greater externalized behavioral problems (e.g. ODD or conduct disorder) and are candidates for medication treatment.

### Persistence in Medication Treatment

Among the patients initially treated with IR–MPH, 46.8% switched their medication to OROS–MPH, and 6.7% switched to ATX. Among the patients prescribed IR–MPH, OROS–MPH, and ATX, 17.8%, 12.6%, and 18.8%, respectively, received prescriptions only once and never returned for drug refill. Although the actual reasons for the premature discontinuation could not be determined from the claims data, this may indicate that numerous ADHD youths or their parents were not well prepared for ADHD medication. Previous studies have indicated that the most frequently reported reasons for medication discontinuation were adverse effects, treatment ineffectiveness, patient attitude or dislike of medication, and social stigma [27, 43]. To diminish the premature discontinuation of medication treatment, a treatment plan integrating adequate psychoeducation should be developed to facilitate cooperation in therapy and the utilization of local community resources.

Among patients prescribed IR–MPH, OROS–MPH, and ATX, the mean time from drug therapy initiation to discontinuation was 539.3, 444.2, and 150.7 days, respectively, whereas the 1-year continuation rates were 49.0%, 61.1%, and 41.2%, respectively. A systematic literature review [26] summarized the data published before 2013, revealing that treatment persistence for stimulants averaged 136 days for youths with ADHD. Recent studies have shown that the rate of medication persistence among ADHD youths over a 12-month period was approximately 30% [28, 29]. Moreover, Lambert et al. [30] reported that overall median treatment duration of stimulants was 1.96 years in a state in Australia. Notably, considerable heterogeneity existed in the definition of treatment discontinuation and length of follow-up among studies. Among the studies that investigated similar topics, the medication gap length defining discontinuation varied from 15 to 180 days [26]; studies defining discontinuation as a longer medication gap typically involved lower discontinuation rates.

Among the three ADHD medications, OROS–MPH had the highest persistence rate at any point during follow-up, whereas ATX had the lowest persistence rate. Studies conducted in the U.S. have indicated that patients initiating extended-release MPH treatment formulations had a significantly longer mean estimated duration of treatment compared with patients initiating IR–MPH treatment [44, 45]. Some clinical data have suggested that OROS–MPH facilitates easy administration to patients, reducing stigma and improving medication adherence [32, 46]. The present results support these previous findings. Although ATX is also a long-acting medication with a tolerable side effect profile, it often requires approximately 6 to 8 weeks to produce pharmacological effects [33]. We propose that early discontinuation of ATX might be associated with the absence of an immediate treatment response. An alternative explanation is that because ATX is the latest drug to enter the market in Taiwan, the follow-up time among ATX users is the shortest.

We found that older age at first prescription significantly predicted early discontinuation of the medications. This finding is generally comparable with those of several previous studies [16, 25, 28, 29]. Longitudinal studies have demonstrated changes in the core symptoms of ADHD as it progresses, particularly decline in hyperactive or impulsive symptoms [6, 47]. Older patients may either achieve symptom remission quicker or may be less likely to respond to medication; therefore, they may discontinue treatment earlier. Another possibility is that older patients are less obedient to parent or physician advice during drug treatment. Moreover, we found that a higher dose of prescribed medication is associated with increased likelihood of early discontinuation. This finding is in line with some previous studies [27, 46]. Compared to patients prescribed lower doses of ADHD medications, their counterparts using higher doses may experience more adverse effects, or have more severe or poorly controlled symptoms. These factors may explain the association between high daily dose and poor adherence of medication. In addition to patient age and dosage, several factors have been associated with early discontinuation of ADHD medications, including doctor specialties, parent educational levels, and patient intelligence quotients [16, 45, 48]. However, these possibilities were not examined in this study. In general, early cessation of ADHD medication may cause inadequate treatment responses and poor outcomes [24]. Ensuring that patients receive sufficient treatment time to achieve functional remission is crucial [27].

### Limitations

This study had several limitations. First, certain clinical information at the personal level (such as the ADHD symptom severity, socioeconomic status, reason for initiating or discontinuing pharmacotherapy, treatment effects, adverse effects, and prognosis) was not provided in the claims data. Therefore, we could not determine whether or how the aforementioned factors affected ADHD pharmacotherapy. Second, the three medications entered the market in Taiwan at various times: IR–MPH on March 1, 1995; OROS–MPH on September 1, 2004; and ATX on May 1, 2007. Therefore, the patients who discontinued IR–MPH treatment before 2004 did not have the opportunity switch to OROS–MPH or ATX. Moreover, a direct comparison of discontinuation factors for MPH and ATX may be unequal. Third, the study employed reimbursement data, and the diagnoses of ADHD were not validated using structural diagnostic instruments; they were identified on the basis of only ICD codes. In addition, the treatment gap (i.e., drug holiday) and combination therapy were not analyzed in this study [49]. Furthermore, a number of patients who switch from IR–MPH to OROS–MPH or ATX may eventually change their drug therapy back to IR–MPH [50]. These particular patient groups were not separately analyzed in this study. Finally, this study was restricted to evaluating the prescription patterns of IR–MPH, OROS–MPH, and ATX because they were the only three drugs approved for ADHD treatment in Taiwan before 2011. The definition of drug discontinuation in this study was 180 days, and this definition might differ from that used in previous international studies. Our results may not be generalizable for other ADHD medications in other countries.

## Conclusion

This nationwide study revealed that IR–MPH was the most frequently prescribed drug for treating ADHD in Taiwan between January 2000 and December 2009. Drug therapy persistence was highest among OROS–MPH-treated patients and lowest among ATX-treated patients. Male sex and neuropsychiatric comorbidities were associated with being prescribed one of the medications. Older age at first prescription and higher doses of prescribed medications significantly predicted early discontinuation of drug therapy. The results provide insight into the delivery of pediatric mental health services and have crucial implications for ADHD medication treatment in real clinical settings. The underlying reasons and clinical outcomes associated with initiation and persistence of ADHD medication treatment warrant further investigation.
